# The “Survivor Peptide” Hypothesis: Structural Resilience and Immunological Persistence of Food Allergens in the Gut–Mammary Axis

**DOI:** 10.3390/nu18111757

**Published:** 2026-05-30

**Authors:** Madalina Coman-Stanemir, Mariana Catalina Ciornei, Cristina Burtescu, Ioana Raluca Papacocea

**Affiliations:** 1 Department of Medical Physiology, Faculty of Medicine, Carol Davila University of Medicine and Pharmacy, 020021 Bucharest, Romania; 2 Independent Researcher, 060011 Bucharest, Romania

**Keywords:** survivor peptide, molecular resilience, gut–mammary axis, structural resilience, IgE epitopes, molecular modeling, protein stability, entero-mammary pathway

## Abstract

**Background:** The translocation of diet-derived antigens from the maternal intestine to breast milk represents a primary gateway for neonatal immune priming, yet the structural basis for why certain proteins survive this transit while others do not remains poorly understood. This review introduces the “Survivor Peptide” hypothesis, proposing that specific food allergens possess intrinsic “stability architectures” that enable them to resist maternal digestion and navigate the gut–mammary axis to reach the infant in an immunologically active form. **Methods:** We analyzed the current literature regarding the detection and structural characteristics of food allergens in human milk. Integrating evidence from 26 major sources, we performed an in silico structural analysis of five representative “survivor” proteins: Gal d 1 (egg white), Bos d 5 (cow’s milk), Gal d 6 (egg yolk), Tri a 19 (wheat), and tropomyosin (Der p 10-mite/shellfish). High-resolution 3D models were retrieved from the Protein Data Bank and AlphaFold2, and then visualized in UCSF ChimeraX to map stability anchors, including disulfide bonds and hydrophobic clusters, against solvent-accessible IgE-binding epitopes. **Results:** We identified and categorized allergens into distinct Molecular Resilience Architectures: the “Covalent Cage” (Gal d 1), defined by dense disulfide stapling, the “Glycoprotein Shield” (Gal d 6), utilizing yolk-matrix structural anchors, the “Topological Shield” (Bos d 5), characterized by a stable β-barrel, and “Coiled-Coil Rigidity” (Der p 10). These frameworks protect large, immunogenic fragments that maintain the spatial arrangement required for IgE cross-linking. **Conclusions:** Allergen persistence in the gut–mammary axis is dictated by a protein’s intrinsic structural architecture. Identifying these stability fingerprints provides a unified theory for allergen persistence and offers a path for refining component-resolved diagnostics and neonatal oral tolerance strategies.

## 1. Introduction

Establishing early life oral tolerance depends fundamentally on a highly coordinated maternal–neonatal immunological dialog, wherein dietary antigens successfully traverse the maternal gut to populate the breast milk matrix. This entero-mammary pathway enables the selective transfer of food-derived proteins and peptides across the gut barrier and into mammary secretions, where they are encountered by the developing immune system in a uniquely tolerogenic context. Increasing evidence implicates both paracellular leak and highly regulated transcellular routes, including trafficking via epithelial endocytosis and immune cell shuttling, as contributors to this process. Among the molecular mediators, the neonatal Fc receptor has emerged as a key player by binding IgG–antigen complexes at acidic pH and recycling them across epithelial barriers, thereby chaperoning otherwise labile dietary proteins from the maternal gut lumen into the circulation and ultimately into breast milk [1,2,3].

A striking feature of these breast milk-borne food antigens is their extraordinarily low concentration, often in the nanogram- to picogram-per-milliliter range, which nevertheless suffices to engage high-affinity IgE on effector cells. This “dose–potency paradox” suggests that only a subset of dietary proteins possess the intrinsic structural features required to survive gastrointestinal processing, resist complete proteolysis, and remain capable of cross-linking IgE after complex trafficking. Rather than functioning as random nutritional debris, these antigens behave as highly efficient templates for immunological recognition. In this context, we introduce the “Survivor Peptide” framework to conceptualize how specific structural and biophysical attributes endow certain food allergens with disproportionate immunological impacts along the entero-mammary axis. Within this framework, Survivor Peptides are defined as proteolytic fragments or structural motifs that persist through the combined stresses of food processing, digestion, epithelial transport, and breast milk secretion while retaining their capacity to bind IgE.

## 2. Materials and Methods

The methodological framework of this study centers on a comprehensive synthesis of current literature regarding the presence, structural integrity and immunological activity of food allergens in human milk. Studies were identified through an iterative search of the PubMed, Scopus and Web of Science databases, focusing on the intersection of maternal dietary intake, gastrointestinal proteolysis and the entero-mammary transport of proteins. Databases were queried using the following Boolean search string: (“food allergens” OR “dietary antigens”) AND (“breast milk” OR “human milk”) AND (“gut–mammary axis” OR “translocation”) AND (“epitope stability” OR “protease resistance”). Inclusion criteria required studies documenting the identification of intact or immunologically reactive dietary proteins in human milk using specific detection methods, namely mass spectrometry (LC-MS/MS), enzyme-linked immunosorbent assays (ELISAs) and Western blotting. The clinical outcomes tracked included neonatal sensitization patterns, atopic dermatitis development, and the induction of oral tolerance. A total of 342 records were initially retrieved; following duplicate removal and strict title/abstract screening against our inclusion criteria, 26 major clinical and experimental sources directly capturing structural profiles in maternal transmission were selected for critical analysis.

To complement this synthesis and explore the biophysical basis of the “Survivor Peptide” hypothesis, five primary proteins identified in the literature as highly persistent—Gal d 1, Gal d 6, Bos d 5, Der p 10, and Tri a 19—were selected for in silico structural mapping. High-resolution atomic coordinates were retrieved from the RCSB Protein Data Bank for Bos d 5 (PDB: 3NPO) and Gal d 1 (PDB: 1CHO). To evaluate global and regional structural confidence metrics, full-length computational models were retrieved from the AlphaFold Protein Structure Database using their respective UniProt identifiers: Gal d 1 (UniProt: P01005), Gal d 6 (UniProt: P02843), Der p 10 (UniProt: O18484) and Tri a 19 (UniProt: Q68A36) [4]. Structural confidence and regional intrinsic flexibility were quantified using the official global average predicted Local Distance Difference Test (pLDDT) extracted from each database entry. Model confidence varied strictly according to the native topological features of each archetype, ranging from highly rigid folds with very high confidence tiers, such as Der p 10 (global average pLDDT: 94.75) and Gal d 6 (global average pLDDT: 74.15), to highly disordered, dynamic storage frameworks characterized by a very low confidence baseline, specifically Tri a 19 (global average pLDDT: 31.33).

All molecular rendering and surface topology analyses were conducted in UCSF ChimeraX version 1.11 [5]. To ensure thematic consistency across the structural algorithms, a standardized visual nomenclature was applied: the primary protein backbone was rendered in steel blue/gray, internal stability anchors (hydrophobic clusters and disulfide bridges) were highlighted in yellow, and experimentally validated linear and conformational IgE-binding epitopes were projected onto the solvent-accessible surface in magenta. This approach allows for the integration of documented clinical evidence with a high-resolution analysis of molecular resilience.

## 3. The Gut–Mammary Axis: Mechanics of Allergen and Microbe Translocation

The presence of diet-derived allergens in human milk is recognized as a complex physiological phenomenon that intersects with maternal systemic health and the microbiome [6,7,8,9]. This entero-mammary pathway involves the coordination of intestinal barrier modulation, immune cell trafficking and selective epithelial transport, though the ultimate immunological consequence for the infant remains a field of ongoing investigation [7,8,9,10,11,12].

### 3.1. The Entero-Mammary Pathway: Cellular and Molecular Shuttling

The maternal gut serves as the primary gateway for both allergens and microbial components [6,7,10,13]. Transcellular and immune-complex transfer represents a significant route for dietary proteins as antigens can be transported across enterocytes via the neonatal Fc receptor (FcRn) when complexed with IgG [8,9,10,11,12]. This process may preserve the structural integrity of specific protein fragments, potentially facilitating their passage into systemic circulation [10,12,14]. In parallel to this, immune cell shuttling provides a secondary transport mechanism where dendritic cells and macrophages in the maternal gut sample luminal antigens and bacteria [10,13]. These cells migrate via the lymphatic system and blood to deliver these components to mammary tissue, a process closely linked to the IgA homing axis, where gut-derived IgA plasmablasts home in on the mammary gland via a CCL28–CCR10-dependent mechanism [8,9,10,13].

The translocation of luminal material is not limited to allergens as recent work on gastro-enterogenic mastitis illustrated how gut barrier damage allows bacterial products and microbiota to reach the mammary gland, highlighting the role of systemic inflammation and dysbiosis in modulating axis permeability [7,15,16].

### 3.2. The Dose–Potency Paradox: Biological Activity at Nanogram Scales

A significant challenge in breastfeeding research is the dose–potency paradox, where nearly undetectable concentrations of allergens retain measurable biological activity [9,12,17,18,19]. Effector potency at trace levels is evident in studies of peanut allergens (Ara h 2), where picogram to low nanogram concentrations remain capable of inducing IgE cross-linking in functional assays [17,18,19,20].

This biochemical potential correlates with clinical reactivity in exclusively breastfed infants, with documented symptomatic reactions to trace levels of egg, milk, and peanut proteins [9,12,17,19]. When analyzed alongside population eliciting doses, these trace exposures often fall within the reactive range for highly sensitive infants, though it is currently unclear whether such low-dose exposure primarily drives sensitization or promotes oral tolerance [9,12,17,19,21].

### 3.3. Barriers to Entry: Selective Permeability and Microenvironmental Context

The efficiency of translocation is governed by the maternal intestinal barrier and its tight junction regulation, which involves pore and leak pathways [7,10,22,23]. However, barrier dysfunction and systemic inflammation can create a “leaky gut” state, which is influenced by diet, exercise, chemotherapy and the gut microbiota, which enhances the paracellular flux of allergens and microbial products [6,7,15,16,22,23]. In allergic individuals, this is further exacerbated by IgE/CD23-facilitated transcellular transport [9,10,24].

The journey from gut to blood to mammary gland is completed by shifts in mammary epithelial permeability during early lactation or pathology, which modulate the transfer of dietary antigens into milk [9,12]. While the co-delivery of resilient protein fragments and immunomodulators like TGF-β suggests a pathway for immune programming [8,9,10,12], the “Survivor Peptide” concept must be viewed with caution. Crucially, structurally resilient dietary peptides act as targets for infant immune recognition but do not determine the timing of clinical symptoms. Symptom onset and evolution instead reflect the underlying immune endotype. Thus, surviving dietary peptides provide the structural antigenic template, whereas clinical kinetics are dictated by the recipient’s immunologic endotype and effector pathways [25,26,27]. The scientific community continues to study whether these mechanisms evolved specifically for allergen transfer and oral tolerance or are secondary effects of broader nutrient and microbial transport systems [6,7,8,9,10,12].

### 3.4. Protein Stability and Maternal–Infant Immune Programming

The transition from maternal dietary intake to neonatal immune priming is strongly shaped by which dietary proteins survive digestion, cross the maternal gut, and appear in breast milk in an immunologically active form. These structurally resilient proteins and peptides act not as random degradation products, but as antigenic templates that can contribute to tolerance or allergy in the infant.

#### 3.4.1. Protein Stability and Antigen Transfer

Human breast milk may contain bovine milk peptides derived from the mother’s diet, which predominantly consist of fragments of bovine milk proteins such as β-lactoglobulin and caseins [28,29]. Mass-spectrometry work has shown dozens of distinct bovine peptides in milk, with several β-lactoglobulin-derived peptides present at significantly higher levels in allergic compared to non-allergic mothers, indicating that dietary β-lactoglobulin can cross the intestinal barrier and be secreted into milk [28,29]. Reviews confirm that breast milk can carry multiple food allergens (egg proteins, peanut Ara h 2, cow’s milk proteins), generally at very low (ng–pg/mL) levels, and that secretion is highly variable between mothers, allergens, and time points [12,18,29,30]. For peanut, intact or near-intact Ara h 2 with IgE-crosslinking capacity has been detected in human milk, with higher peak levels in atopic mothers and substantial inter-individual heterogeneity [18]. Together, these findings establish a biological precedent for an entero-mammary pathway that selectively transmits structurally stable allergens as “templates” rather than random debris.

#### 3.4.2. Immunological Bioavailability

Across experimental and human work, very low doses of dietary antigens in breast milk have been shown to be immunologically meaningful. Narrative and systematic reviews emphasize that maternal diet directly shapes allergen levels in human milk, that different antigens display distinct kinetics and persistence, and that typical concentrations are in the nanogram-per-milliliter range but can still influence oral tolerance induction [12,30,31,32]. Mechanistically, antigens can be transferred as free proteins/peptides or as immune complexes with IgA or IgG. Mouse models show that food allergens transferred in IgA or IgG immune complexes via milk can induce robust oral tolerance, expanding antigen-specific regulatory T cells and preventing anaphylaxis and IgE production in offspring [11,33]. Human breast milk containing allergen–IgG immune complexes likewise induced tolerance in humanized mice [11]. Reviews integrating these data propose that breast milk delivers a structured, low-dose antigenic library, whose impact depends on the dose, molecular form (free vs. immune complex), and accompanying immune factors, explaining why trace exposures can be either tolerogenic or, in some conditions, sensitizing [31,32,34,35,36]. This aligns closely with the idea of epitopic bioavailability: structurally preserved epitopes, not bulk protein quantity, determine functional potency.

#### 3.4.3. Impact of Food Processing on Allergen Transfer

Food processing further modulates which epitopes survive to reach breast milk. Narrative reviews on exogenous allergens in human milk highlight that the mode of antigen consumption, including cooking, changes the allergenicity of proteins that later appear in milk [30]. Heat treatment and other processing of cow’s milk proteins are known to alter casein and whey protein structures, digestibility and IgE binding, which in turn may modify the allergenic profile of residual peptides detected in human milk and the balance between tolerance and sensitization in infants [8,29]. Pasteurization and other industrial treatments of donor milk may not only change endogenous human milk factors but could also influence the form and immunogenicity of residual cow’s milk antigens, potentially affecting allergy risk in recipients [29]. These observations support the notion that protein-specific “stability architectures” (a combination of intrinsic structural resilience and extrinsic processing) govern which allergen fragments navigate the maternal gut, circulation, and mammary gland.

#### 3.4.4. Maternal Diet Patterns and Infant Outcomes

Despite the capacity of maternal diet-derived proteins to reach milk, population-level data and expert reviews agree that maternal avoidance diets during pregnancy or lactation do not prevent food allergies and should generally be discouraged for primary prevention [34,35]. Instead, continued breastfeeding together with a nutritionally adequate, non-restrictive maternal diet is viewed as important for healthy immune programming, allowing for controlled exposure to dietary proteins that may favor tolerance rather than sensitization [34,35,36]. Reviews emphasize that maternal health, diet quality, allergy status and other exposome factors shape breast milk composition, ranging from antigens to immunoglobulins, cytokines, oligosaccharides, metabolites, and microbiota, which collectively influence infant gut colonization, barrier function and immune development, including allergy risk [31,32,36,37,38,39,40,41]. Some observational data suggest that higher maternal intake of specific allergenic foods (such as cow’s milk) correlates with increased allergen-specific antibodies in milk and may support tolerance processes, whereas strict avoidance reduces allergen-specific IgG titers [42]. Crucially, this reduction in maternal antibody titers suggests that strict avoidance diets do not merely fail to prevent atopy, but may paradoxically increase the risk of infant food allergy development. Without maternal dietary exposure, the absence of trace ‘survival fragments’ halts the formation of protective allergen–IgG immune complexes. Deprived of these vehicles, the nursing infant loses the critical FcRn-mediated signaling required to prime regulatory T-cell (Treg) networks in the gut, leaving them vulnerable to unbuffered environmental sensitization. Overall, maternal diet functions less as a simple on/off switch for allergen transfer and more as a tunable template that conditions both the antigen dose and the immunological “packaging” (e.g., immune complexes) encountered by the infant.

While maternal dietary patterns and the mechanics of the gut–mammary axis establish the physiological context for antigen exposure, the ultimate fate of dietary proteins is dictated by their own molecular architecture. To understand why certain proteins persist as immunogenic signals while others are degraded into nutritional debris, we must define the specific molecular and immunological determinants that govern protein allergenicity and structural resilience.

## 4. Molecular and Immunological Determinants of Protein Allergenicity

A protein is defined as an allergen based on its dual capacity to (1) induce a loss of oral tolerance, leading to the production of allergen-specific IgE (sensitization), and (2) subsequently cross-link these IgE molecules on the surface of effector cells to trigger physiological degranulation (elicitation).

### 4.1. Structural and Biophysical Prerequisites for Allergenicity

The propensity of a protein to act as an allergen is governed by a complex interplay of immunologic requirements and intrinsic biophysical stability. At the molecular level, an allergen must possess a requisite density of T-cell epitopes capable of being processed and presented via Major Histocompatibility Complex class II (MHC II) and B-cell epitopes for IgE binding. These epitopes drive the polarization of CD4^+^ T cells toward a Th2 phenotype, characterized by the secretion of IL-4 and IL-13, which are the primary drivers of B-cell isotype switching to IgE [43,44]. Epitopes typically consist of 6–15 amino acid residues and may be classified as linear (sequential) or conformational (discontinuous), with the latter being highly dependent on the protein’s native 3D fold [45,46]. Consequently, the preservation of these immunological signals throughout the digestive process is intrinsically linked to specific biophysical features and the “Stability Gradient” that defines a protein’s resistance to denaturation and proteolysis:

- Proteolytic and Thermal Resilience: To maintain immunogenicity, allergens must demonstrate significant resistance to gastroduodenal pH levels and endosomal proteolysis. This stability ensures that intact proteins or large, “nicked” immunogenic fragments reach antigen-presenting cells (APCs). This resilience is frequently mediated by high disulfide bond densities, compact globular folds (e.g., the “Covalent Cage”), or the binding of protective ligands [47,48,49,50]. It determines the nutritional bio-accessibility of the allergen, ensuring that immunogenic fragments remain intact after maternal processing.
- Surface Topology and Charge: Allergenic potential is enhanced by high aqueous solubility and specific surface characteristics, such as negatively charged, solvent-exposed protruding regions that facilitate high-affinity IgE docking [47,50,51]. These regions frequently feature distinct clusters of charged residues, forming positive (Lys/Arg-rich) or negative (Asp/Glu-rich) electrostatic hotspots that optimize high-affinity docking with IgE complementarity-determining regions [46]. These docking sites are highly hydrophilic, less lipophilic and anchored onto underlying structurally stable scaffolds that prevent conformational collapse [46,51]
- Molecular Motifs and Modifications: Conserved structural motifs and specific amino-acid usage patterns often differentiate allergenic families from non-allergenic homologs [52,53,54]. Furthermore, post-translational modifications, particularly N-glycosylation, and interactions within the food matrix can act as adjuvants, enhancing protein uptake or shielding epitopes from degradation [44,50,52].

### 4.2. The IgE–Allergen Interactome: From Sensitization to Elicitation

The clinical manifestation of allergy is a two-stage process mediated by the high-affinity IgE receptor, FcεRI. The sensitization phase takes place during the initial encounter, when the allergen is internalized by APCs, processed into peptides, and presented to naive T cells. The resulting Th2-biased cytokine environment induces B cells to produce allergen-specific IgE [47,55]. These IgE molecules circulate and subsequently bind via their Fc region to the α-chain of FcεRI, which is primarily expressed on the surface of mast cells and basophils [46,56,57].

During the effector phase (elicitation and degranulation) that occurs upon subsequent re-exposure, the allergen must function as a multivalent scaffold. It is not the binding of IgE alone, but the multivalent cross-linking of at least two adjacent FcεRI-bound IgE molecules that initiates the signaling cascade [46,55,58,59]. This spatial bridging triggers receptor aggregation, activating intracellular tyrosine kinases, which leads to the rapid exocytosis of preformed mediators (e.g., histamine) and anaphylaxis [57,60].

The potency of this elicitation phase is critically constrained by spatial and geometric requirements for IgE receptor cross-linking: the density, accessibility and architectural arrangement of surface epitopes must fall within a functional spatial range (typically spanning approximately 15–20 Å) to permit the simultaneous bridging of adjacent, cell-bound FcϵRI complexes [48,51,61]. Allergenicity is a multifaceted phenomenon that extends beyond simple IgE immunoreactivity. Effector activity depends fundamentally on a protein maintaining its structural integrity or nesting within a protective food matrix to present a specific spatial landscape of T-cell and B-cell epitopes [47,51,62]. This architectural arrangement is essential to facilitate the multivalent cross-linking of FcϵRI-bound IgE on effector cells, a process strictly governed by epitope spacing, valence and antibody binding density rather than raw serum IgE titers [63,64]. While systemic allergens are typically characterized by intrinsic structural stability (such as dense disulfide coupling), less stable or easily denatured proteins can leverage food-matrix-mediated protection, such as lipid encapsulation or carbohydrate shielding, to delay proteolysis and safely preserve their conformational epitopes during gastrointestinal transit [65,66]. Conversely, highly labile allergens lacking these protective barriers generally collapse into linear sequences, limiting their activity to localized mucosal reactions [64]. Thus, the clinical allergenicity of a dietary protein is an emergent property shaped by the interaction between its baseline structural stability, the shielding by the food matrix, and the recipient’s immune system downstream effector pathways.

Consequently, maternal dietary restriction fails to protect the infant. Instead, it deprives the entero-mammary pathway of these structurally resilient templates, shifting the maternal–fetal axis away from regulated tolerance toward unbuffered mucosal exposure.

## 5. The Stability Gradient: Classification of Molecular Resilience Architectures

The ability of a dietary protein to navigate the maternal gastrointestinal tract and emerge in breast milk as a functional immunological signal is highly dependent on the intrinsic structural resilience of the protein matrix. This ability behaves as a conceptual ‘Stability Gradient’: it depends on a hierarchy of structural features that strongly influences the protein’s capacity to resist deep proteolysis. While host physiological factors (such as maternal digestive kinetics and epithelial barrier integrity) dictate systemic exposure, the structural classification detailed below outlines the molecular features that favor survival over complete degradation into nutritional debris. Experimental work and reviews showed that disulfide bonds, tightly packed secondary structures and hydrophobic domains can confer resistance to gastrointestinal digestion while preserving IgE-binding epitopes, whereas more flexible, unstructured proteins are generally more labile [67,68]. While the primary amino-acid sequence provides the necessary T-cell and B-cell epitopes, it is the higher-order architecture that dictates whether these signals remain recognizable to the infant immune system by maintaining surface-exposed, conformational IgE epitopes [51,69].

To systematically analyze these survivors, we propose a classification based on Molecular Resilience Architectures. These algorithms represent distinct biophysical strategies, ranging from dense covalent “stapling” to topological shielding and compact fold architectures, that allow allergens to preserve native-like 3D geometry despite digestive and processing stress [67,68]. Conformational epitope mapping and structural studies demonstrate that IgE binding typically targets protruding, surface-exposed regions on a folded protein and depends on the preservation of specific spatial arrangements of residues rather than on linear sequence alone [51,69]. By categorizing allergens through these structural lenses, we can move beyond food-specific descriptions toward a unified theory of protein persistence and epitope preservation in maternal–infant nutrition. A comparative overview of these molecular architectures, their digestive stability, and their clinical implications is synthesized in Table 1.

However, a protein’s macro-structural stability presents a mixed and sometimes contradictory picture when used as a sole predictor of clinical allergenicity [95]. The limitations of a strict, binary focus on intact protein resistance are clearly illustrated by the major allergens from cashew (*Anacardium occidentale*). While the 2S albumin Ana o 3 exhibits profound, intrinsic resistance to gastroduodenal proteolysis, its companion cupin allergens, the 7S globulin Ana o 1 and the 11S globulin Ana o 2, are readily degraded under standard proteolytic conditions. Despite these drastically different degradation rates, all three remain highly potent clinical allergens [96].

This apparent paradox highlights a vital evolutionary nuance: ‘readily digested’ macromolecules often do not undergo complete transformation into inert amino acids. Instead, their rapid initial cleavage routinely unzips the tertiary structure to release small, highly stable and deeply nested “Survivor Peptides” that retain full IgE-binding functionality and conformational reactivity [96,97].

### 5.1. The “Covalent Cage”: Disulfide-Stapled Resilience

The persistence of egg allergens during gastrointestinal digestion is shaped by defined structural features rather than random degradation. Within the “Survivor Peptide” framework, allergens that maintain their 3D integrity are those with structures reinforced by high-density covalent cross-links, which prevent the spatial collapse of IgE epitopes.

#### 5.1.1. Gal d 1: The Kazal-Type “Covalent Cage”

Ovomucoid (Gal d 1) is a 28 kDa glycoprotein that makes up ~10–11% of egg whites and is the major egg allergen; it is strongly associated with persistent egg allergy [74,98]. As a member of the Kazal-type serine protease inhibitor family, its biological function in the avian egg is to act as a potent trypsin inhibitor, protecting the developing embryo from premature proteolytic degradation [98,99]. While its theoretical mass is 28 kDa, its high carbohydrate content often causes it to appear significantly larger (35–40 kDa) in electrophoretic analysis, suggesting a glycosylation shield that complements its inhibitory activity [67,100]. This inhibitory function is a critical component of its allergenicity. By actively subverting digestive enzymes, Gal d 1 extends its own half-life within the gastrointestinal tract, effectively behaving as a self-protecting “Covalent Cage” allergen and increasing the probability of its intact transfer across the intestinal barrier [100,101]. Experimental digestion assays confirm that while Gal d 1 is susceptible to limited cleavage by pepsin, the resulting peptide fragments (or “nicked” units) remain immunologically active and structurally tethered by internal disulfide bridges, resisting complete degradation even under simulated gastric conditions [67,71,101,102].

**Figure 1:**
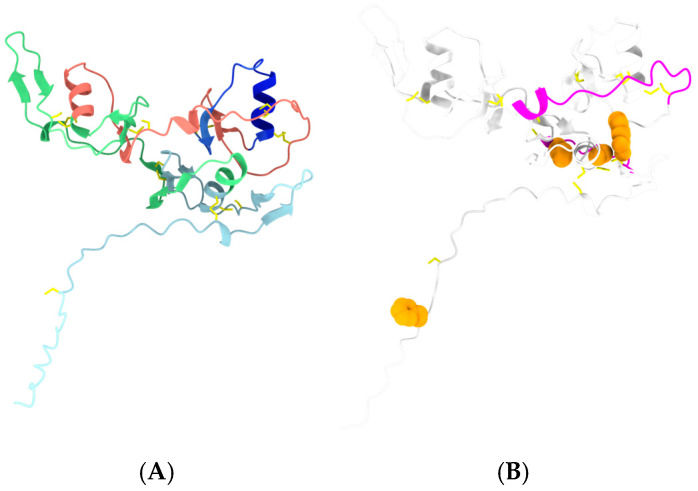
The structural-immunological axis of Gal d 1 (ovomucoid) resilience. (**A**) The “Covalent Cage”: 3D architecture highlighting the structural redundancy of the three tandem Kazal domains (Domain I: blue; Domain II: green; Domain III: salmon). Yellow sticks represent the nine conserved disulfide bonds that provide the mechanical rigidity necessary to survive maternal gastrointestinal proteolysis. (**B**) The Immunological Surface: Mapping of verified “Survivor Peptides” and protective motifs. Magenta regions indicate the solvent-exposed, immunodominant IgE-binding epitopes that persist after digestion. Orange spheres highlight *N*-glycosylation sites (N10, N53, N69, N75, N175), which form the “Glycan Shield” that further stabilizes the protein scaffold against enzymatic degradation. The models were generated using the AlphaFold Protein Structure Database (Entry P01005) and visualized in UCSF ChimeraX. Note: Reflecting its highly ordered, rigidly cross-linked tertiary fold, the computational model exhibits a high overall average model confidence (pLDDT) score of 86.31.

The structural resilience of Gal d 1 is anchored in its unique architecture, which consists of three tandem, homologous domains reinforced by nine internal disulfide bonds (three per domain) [70,103,104,105]. This “Covalent Cage” maintains a dense fold dominated by α-helix and β-sheet motifs, with remarkably few random coil regions. Recent molecular dynamics (MD) simulations confirm that these topological constraints allow Gal d 1 to maintain surface-accessible linear B-cell epitopes even under significant environmental stress. To quantify this stability, researchers utilize MD parameters such as Root Mean Square Deviation (RMSD), which measures global structural shifts. The low and stable RMSD of Gal d 1 at temperatures of up to 80 °C demonstrates that the disulfide staples successfully prevent unfolding, though slight atomic deviations begin to occur at 100 °C as the solvent-accessible surface area (SASA) increases [70] (Figure 1). While the Gal d 1 “Covalent Cage” remains remarkably resistant to endogenous human proteases, experimental exposure to the fungal protease Proteinase K (from *Tritirachium album*) demonstrates that its linear IgE-binding epitopes can be disrupted when subjected to non-specific, high-potency enzymatic cleavage [106].

Complementing global analysis, Root Mean Square Fluctuation (RMSF) is used to measure the local flexibility of specific amino acid residues [70,107,108,109]. While the disulfide framework prevents total collapse, MD simulations reveal high fluctuations at specific “hotspots,” namely Aspartic Acid at position 104 (D104) and Glutamine at position 139 (Q139) [70,103,104,105,110]. These residues act as thermal “hinges” that exhibit increased mobility under extreme stress. Because these hotspots are located within predicted linear B-cell epitopes, their identification is significant and it implies that even if the “Covalent Cage” remains intact, subtle conformational shifts at these sites may modulate the binding affinity of maternal-derived IgE antibodies in the infant gut [70,73,74,111].

Crucial to its survival is the role of the physiological environment. While the covalent cage provides immense physical stability, research indicates that glutathione-mediated reduction in the gut may unlock this structure, exposing specific sequential (linear) epitopes that are otherwise hidden in the native fold [112]. The recognition of these newly exposed linear sequences is a clinical hallmark of persistent egg allergy, suggesting that Gal d 1 remains dangerous even as it begins to unfold [112]. Clinical evidence confirms that approximately one-third of patients recognize purely conformational sites on Gal d 1, suggesting that the survivor unit is often a structurally intact domain rather than a random peptide string [113,114].

#### 5.1.2. Ara h 2: The 2S Albumin “Covalent Cage”

Complementing the avian model of Gal d 1, Ara h 2, the immunodominant 2S albumin from peanut, exemplifies a plant-derived “Covalent Cage.” Ara h 2 is a small protein (~17 kDa) that contains eight cysteine residues that form four conserved disulfide bonds, stabilizing a compact five-helix bundle typical of the prolamin superfamily, and is structurally related to amylase/trypsin inhibitors [80,115]. Reduction of these disulfide bonds causes major loss of the secondary and tertiary structures, confirming that the covalent network is the principal determinant of its structural resilience [115].

The proteolytic stability of Ara h 2 is extreme. In simulated gastric and intestinal digestion, pepsin, trypsin and chymotrypsin generate relatively large, digestion-resistant fragments. Many predicted cleavage sites remain protected by the compact fold and these fragments retain intact IgE-binding epitopes [115,116]. Comparative digestion studies show that Ara h 2 (and Ara h 6) are markedly more resistant to pepsin than Ara h 1 and Ara h 3, with limited proteolysis only evident under reducing conditions, underscoring the critical role of disulfide bonds in maintaining the overall structure [116]. Even after cleavage in exposed loops, the core 3D architecture and allergenic potency are largely preserved so that protease-treated Ara h 2 can still efficiently trigger mediator release from effector cells [115,117]. This highlights how preservation of conformational epitope architecture, rather than an intact primary sequence, underlies its high IgE-cross-linking capacity [115,117,118].

Beyond its intrinsic stability, Ara h 2 also functions as a trypsin inhibitor. Homology and functional assays demonstrate that Ara h 2 belongs to the proteinase/α-amylase inhibitor family and directly inhibits trypsin activity. Roasting further enhances this inhibitory function [80,117]. By both resisting digestion and actively attenuating protease activity, Ara h 2 prolongs its own survival and that of associated digestion-resistant peptides within the food matrix and gastrointestinal tract [67,100,101]. These structural and functional features, together with the documented transfer of Ara h 2 and digestion-resistant peptides into human breast milk in immunologically active form [14,103,104], support its characterization as a highly persistent “Covalent Cage” allergen capable of maintaining potent biological activity even after extensive processing and passage through the maternal gut–mammary axis.

#### 5.1.3. Gal d 2: The Labile Scaffold

Contrasting the extreme resilience of the “Covalent Cage” and “Redundant Scaffold” is Gal d 2 (ovalbumin), a ~45 kDa serpin-like protein that serves as a critical point of comparison in the structural persistence hypothesis. Unlike Gal d 1 and Gal d 5, the 3D integrity of Gal d 2 is reinforced by only one internal disulfide bond (Cys73–Cys120), leaving the majority of its globular architecture dependent on non-covalent hydrophobic packing and hydrogen bonding [63,105].

Fine mapping using overlapping peptides has localized five dominant IgE-binding regions within the Gal d 2 sequence (L38–T49, D95–A102, E191–V200, V243–E248, and G251–N260), and correlated these linear segments to β-sheets, α-helices and turns [76]. However, clinical and serological data indicate that IgE recognition of these linear peptides is less frequent and generally weaker than responses to more structurally constrained egg allergens, implying that ovalbumin allergenicity is primarily driven by conformational epitopes [63,105,106,107].

Within the maternal gastrointestinal tract, the lack of an extensive disulfide network renders Gal d 2 highly susceptible to unfolding under acidic or thermal stress: heating, glycation, and aggregation induce unfolding, cross-linking, and epitope remodeling that substantially alter IgE binding [105,107]. Once this native serpin fold is disrupted, the spatial orientation of its conformational epitopes is lost and the protein is less able to support multivalent FcεRI-bound IgE cross-linking, which is required for a full clinical response [105,107]. Consequently, while Gal d 2 is an immunodominant allergen in raw or lightly cooked egg, its role as a digestion-resistant “Survivor Peptide” in the gut–mammary axis is limited by its structural lability compared with its disulfide-dense counterparts [63,105,107].

In the context of the Covalent Cage–Redundant Scaffold–Labile Scaffold spectrum, Gal d 2 functions as a structurally fragile comparator, illustrating how limited covalent stapling constrains an allergen’s ability to survive the maternal gut and contribute durable ‘Survivor Peptides’ to the gut–mammary axis [108,109].

### 5.2. The Redundant Scaffold and Reversible Memory

#### 5.2.1. Gal d 5: Resistance Through Structural Redundancy

In contrast to the compact cage of Gal d 1, Gal d 5 (serum albumin, α-Livetin) forms a massive, heart-shaped α-helical scaffold built from three domains stabilized by 17 conserved intramolecular disulfide bridges [110]. As a serum albumin, Gal d 5’s core physiological role is to act as a multi-ligand transporter in avian blood, binding fatty acids, hormones, and other small molecules while maintaining colloid osmotic balance [111,112,113]. This transport function demands a highly stable, heart-shaped α-helical scaffold in which 17 conserved intramolecular disulfide bridges lock the three domains around buried hydrophobic pockets, ensuring carrier competence even under fluctuating physiological conditions [112,114,115]. Molecular dynamics and kinetic studies show that this dense S–S network acts as a rigid structural core: the reduction of disulfides markedly destabilizes the secondary structure and distorts the tertiary fold [116,117]. However, MD simulations reveal that only specific disulfide pairs are individually essential, while others can be broken with limited structural impact, providing the protein with built-in redundancy [117,118]. Gal d 5 highlights the emerging role of bioinformatic 3D modeling in predicting persistence. Homology models reveal a diverse landscape of surface-accessible B-cell and T-cell epitopes, suggesting that its globular albumin-like structure may facilitate translocation via protected hydrophobic pockets [80,119].

These 17 internal disulfides are strongly stabilized and do not readily participate in physiological redox cycling. Rather, they act as locked structural staples that define the canonical scaffold [110,116]. This disulfide-encoded structural redundancy means that partial biochemical “nicking” or selective bond reduction during digestion rarely suffices to trigger global unfolding. The albumin fold continues to present its conformational epitope landscape, supporting Gal d 5’s classification as a structurally persistent “Redundant Scaffold” allergen in the Survivor Peptide framework [120,121]. This redundancy ensures that the overall protein fold can withstand the partial biochemical “nicking” encountered during digestion, as seen by the need to cleave sets of disulfides before large-scale unfolding in albumins [117,118]. Mapping studies demonstrate that heat and storage can induce disulfide reshuffling and new inter- and intramolecular links, yet the core resilience of the albumin scaffold remains [105,122]. Under reductive radical stress, recombination of sulfur-centered radicals can create stable aggregates, highlighting the structural centrality of this disulfide network [105]. By maintaining domain-level integrity through this redundant network, Gal d 5 preserves its native compact state and thus the spatial orientation of its conformational epitopes, supporting its potential to act as a structurally persistent, highly immunoreactive allergen [110,116,117,118].

#### 5.2.2. Gal d 4 (Lysozyme): Reversible Unfolding and Native-State Memory

Gal d 4 (lysozyme), a 14.3 kDa antimicrobial enzyme, serves as a prototype for the Refolding Architecture. Its architecture is stabilized by four internal disulfide bonds which, unlike a rigid “Covalent Cage,” permit substantial conformational flexibility and allow for non-native states that still retain elements of native-like order in the unfolded ensemble [123].

Whereas Gal d 1 persists through mechanical rigidity, the survival of Gal d 4 relies on its capacity for reversible unfolding. Biophysical studies show that, under suitable solution conditions, thermally denatured lysozyme can refold to a native-like structure and recover enzymatic activity after heating to near-boiling temperatures, indicating a substantial structural memory of the native state [124,125]. Gal d 4 is structurally robust to extreme pH, but covalent modification (e.g., Au–S gold nanocluster bioconjugation) drives a stable transition from an α-helical, enzymatically active, IgE-reactive state to a perturbed, β-sheet-enriched conformation with reduced activity and IgE binding, highlighting the strong dependence of conformational epitopes on native fold integrity [126]. This structural shift results in a catastrophic loss of both enzymatic activity and IgE-binding capacity, highlighting that Gal d 4’s role as a “Survivor Peptide” is strictly contingent upon its ability to maintain or restore its native fold integrity.

In the context of the maternal–infant axis, Gal d 4 can thus be conceptualized as a “Stealth Survivor”: it may exist in partially unfolded or perturbed states under gastric stress yet retain the intrinsic disulfide-guided memory required to refold into a compact, epitope-competent conformation once it encounters more permissive environments such as the mammary gland or neonatal gut. This behavior distinguishes it from more labile scaffolds (for example, Gal d 2), which lack the disulfide-driven conformational bias needed to reliably recover their three-dimensional architecture once disrupted.

While the “Covalent Cage” and “Redundant Scaffold” rely on dense disulfide networks, other major food allergens utilize distinct algorithms of sequestration and topological shielding to navigate the maternal–infant axis. We next examine the milk-derived survivors that utilize a ‘Lipocalin Calyx’ and ‘Micellar Sequestration’ to maintain immunological recognizability.

### 5.3. Emerging Survivors: Gal d 6 and the Glycoprotein Shield

While the white-derived “Covalent Cage” and “Redundant Scaffold” dominate the clinical landscape, emerging data on Gal d 6 (YGP42) highlight a secondary yolk-derived survivor with distinct persistence characteristics. Gal d 6 is the *C*-terminal fragment of vitellogenin-1 (YGP42), the second egg-yolk allergen to be characterized, and is recognized by IgE in a substantial subset of egg-allergic patients; it is less structurally characterized than the major egg-white allergens [127,128]. Epitope-mapping and immunoinformatics studies further support its status as a bona fide allergen by defining multiple predicted T- and B-cell epitope clusters across its sequence [62,80].

**Figure 2:**
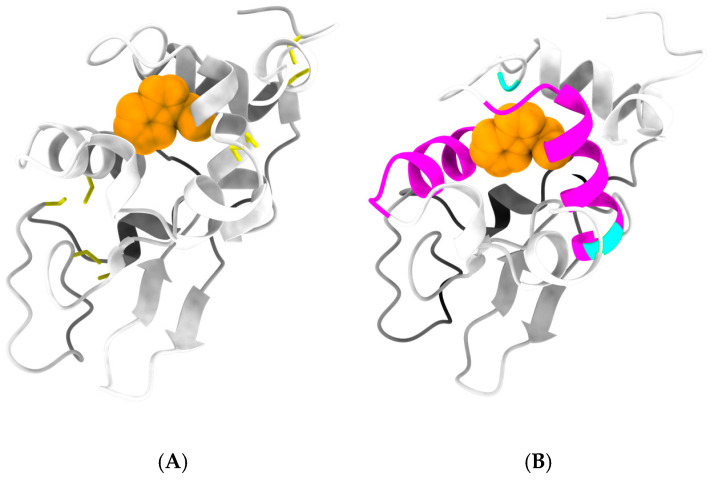
Structural mapping of the “Glycoprotein Shield” in Gal d 6 (vitellogenin-1). (**A**) Structural Framework and Stability Anchors: Three-dimensional model of the *C*-terminal fragment (YGP42) of vitellogenin-1. The protein backbone is rendered in steel blue, with internal hydrophobic regions highlighted in yellow. These regions act as structural “anchors” within the lipid-rich yolk matrix, contributing to the “shield” effect that preserves the protein core. (**B**) Immunological Landscape: Surface mapping of persistent IgE-binding epitopes (rendered in magenta). The visualization highlights the structural resilience of regions AA180–220 and AA240–284, which maintain their conformational integrity despite localized disruptions (e.g., AA262–268) caused by high-pressure thermal processing. The models were generated using the AlphaFold Protein Structure Database (Entry P02845) and visualized in UCSF ChimeraX. Note: Reflecting its combination of stable globular domains interspersed with flexible connecting segments, the computational model exhibits a pLDDT score of 74.15.

Recombinant expression of Gal d 6 in *Escherichia coli* has confirmed robust IgE reactivity, with four linear B-cell epitopes that were identified experimentally [128,129]. High-pressure assisted thermal processing significantly reduces IgE/IgG binding and degranulation, but does not abolish reactivity, indicating that a baseline allergenic core persists after industrial-scale stress [129,130]. Circular dichroism and molecular dynamics show substantial alterations in secondary structure, particularly in regions AA180–220 and AA240–284, with disruption focused on the AA262–268 epitope, implying that other epitopic regions and glycoprotein features sustain residual allergenicity [80,129] (Figure 2).

Positioning Gal d 6 as a “secondary yolk survivor” is consistent with its origin as a vitellogenin-derived yolk glycoprotein within a lipid-rich, protein-dense environment that harbors several immunogenic yolk components, including YGP42 and YGP40 [62,128]. In this context, the vitellogenin-based glycoprotein scaffold and yolk matrix can be viewed as a glycoprotein shield that favors partial epitope preservation and supports the contribution of Gal d 6-derived sequences to the persistent “Survivor Peptide” pool within the maternal–infant axis.

### 5.4. Supramolecular Sequestration: The Milk Allergen Architecture

In parallel with Gal d 1 and Ara h 2, cow’s milk allergens implement two complementary persistence strategies in the maternal gut–mammary axis: a lipocalin “Topological Shield” for whey, and a micellar “Linear Persistence” strategy for caseins, which are both tightly coupled to nutrient delivery.

Bos d 5: The Lipocalin “Topological Shield”

**Figure 3:**
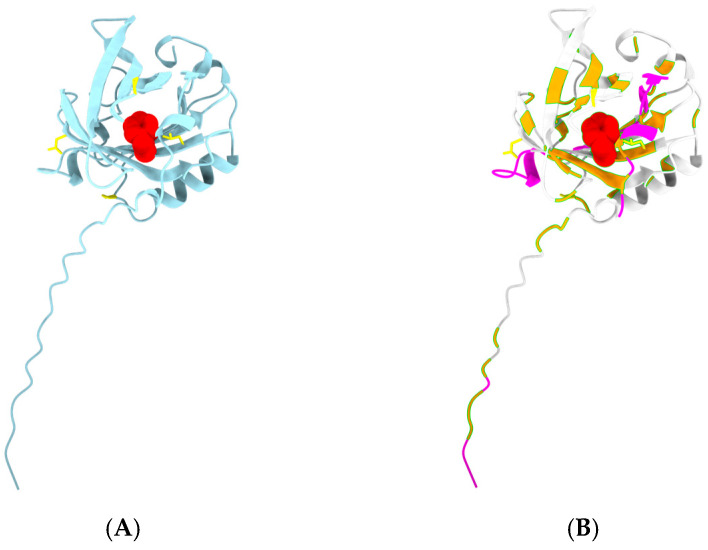
The hydrophobic calyx and conformational resilience of Bos d 5. (**A)** Structural Architecture: 3D model of β-lactoglobulin (Bos d 5) illustrating the 8-stranded antiparallel β-barrel (light blue) that forms the central “calyx” or hydrophobic pocket. Yellow sticks represent the two internal disulfide bonds (C66–C160 and C106–C119) that maintain the “closed” conformation. The red sphere highlights the free thiol (C121), a critical hotspot for heat-induced aggregation and thiol-disulfide exchange. (**B**) The Immunological Surface: Mapping of persistent linear epitopes (magenta) at the *N*-terminus and the *C*-terminal loop (residues 125–135). The orange regions represent the internal hydrophobic core, which effectively “shields” the protein’s interior from aqueous gastrointestinal proteases. The models were generated using the AlphaFold Protein Structure Database (Entry P02754) and visualized in UCSF ChimeraX. Note: Reflecting its highly compact, rigidly organized classic globular lipocalin fold, the computational model exhibits an exceptionally high overall pLDDT score of 91.95.

β-Lactoglobulin (Bos d 5) is the predominant whey allergen and a member of the lipocalin/calycin superfamily; it comprises a compact eight-stranded β-barrel that forms a hydrophobic calyx for fatty acids and other ligands. Ligand binding and the β-barrel topology stabilize the secondary and tertiary structures, placing Bos d 5 among the disulfide- and barrel-stabilized allergens that resist gastrointestinal digestion and preserve IgE epitopes [54,131] (Figure 3).

Simulated infant digestion coupled with peptidomics showed that Bos d 5 is only partially cleaved by gastric and intestinal enzymes, yielding a limited set of digestion-resistant peptides that retain strong IgE binding (KD 17–279 nM) and 40–80% of the allergenic potency of the intact protein [131]. In multistep static digestion of pasteurized, UHT and dried milks, Bos d 5 is gradually degraded, yet short (3.4–5.0 kDa) peptides survive all digestive phases and cross an Ussing chamber model. Ten such peptides map to known sequential epitopes, including β-LG 92–100, 125–135/138 and 149–162, and α-LA 63–79 and 80–93, confirming that digestion-resistant fragments still carry clinically relevant IgE determinants [131,132].

Processing remodels but does not neutralize this mechanism. Industrial heating and Maillard-type glycation denature and aggregate Bos d 5, but digestion-resistant epitope peptides are still recovered, particularly from pasteurized and UHT milks [83]. In yogurts, lactic fermentation preferentially hydrolyses Bos d 5 and Bos d 4, sharply reducing IgE and IgG binding while leaving an abundance of caseins. Mass spectrometry of low-allergenicity yogurt identified Bos d 5 peptides that had lost multiple IgE epitopes yet preserved internal T-cell epitopes with potential tolerogenic function [133,134,135,136].

Clinically, broad IgE recognition of multiple Bos d 5 and Bos d 4 linear epitopes is associated with persistent cow’s milk allergy, whereas children likely to outgrow this allergy recognize fewer Bos d 5 epitopes and essentially no α-LA epitopes [132]. Together, these data justify describing Bos d 5 as a lipocalin “Topological Shield” allergen: a disulfide-anchored β-barrel calyx that resists digestion, sheds epitope-dense Survivor Peptides across processing conditions, and stratifies clinical persistence according to epitope spread [43,54,70,132,133].

Caseins (Bos d 8–12): Micellar “Linear Persistence”

In contrast to the compact lipocalin cage, caseins (αs1-, αs2-, β- and κ-casein) are intrinsically disordered phosphoproteins that assemble with amorphous calcium phosphate into large, dynamic micelles. This fuzzy, mineral-dense complex lacks a rigid globular core but presents extended stretches of solvent-exposed sequence, many of which correspond to IgE epitopes [51,67].

Digestomics of whole milk and dairy products shows that ~97% of short digestion-resistant peptides (modal length: ~10–11 amino acids) derive from caseins and extensively overlap with mapped IgE epitopes on αs1-, αs2-, β- and κ-casein [132]. In vitro GI digestion of pasteurized, UHT and dried milks followed by transepithelial transport reveals that α-casein peptides 25–32, 84–90 and 125–132 persist in the mucosal compartment, alongside β-LG and α-LA epitope peptides [83]. These casein-derived SDRPs can aggregate into non-covalent complexes that retain IgE reactivity and provoke skin-prick responses, despite their small size [132].

Fine epitope mapping in αs1-casein has identified multiple sequential and conformational IgE regions, some of which are selectively recognized in persistent versus transient CMA [51,67]. Fermentation and targeted proteolysis can preferentially cleave within such hotspots, destroying IgE epitopes while sparing T-cell epitopes and generating bioactive peptides with antihypertensive, antioxidant or antimicrobial functions [133].

Clinically, children with persistent CMA have immune systems that recognize numerous linear casein epitopes, often in combination with extensive whey-epitope recognition, whereas the immune systems of patients likely to tolerate milk recognize fewer and less diverse sequential epitopes [43,132]. The casein micelle therefore embodies a “Micellar Sequestration/Linear Persistence” algorithm: intrinsically disordered chains and mineral packing shield long epitope clusters from complete proteolysis, releasing overlapping, aggregation-competent SDRPs that remain IgE-legible and contribute to sustained allergenicity along the gut–mammary axis [43,70,132,133].

### 5.5. Coiled-Coil Rigidity: The Tropomyosin “Supercoil”

**Figure 4:**
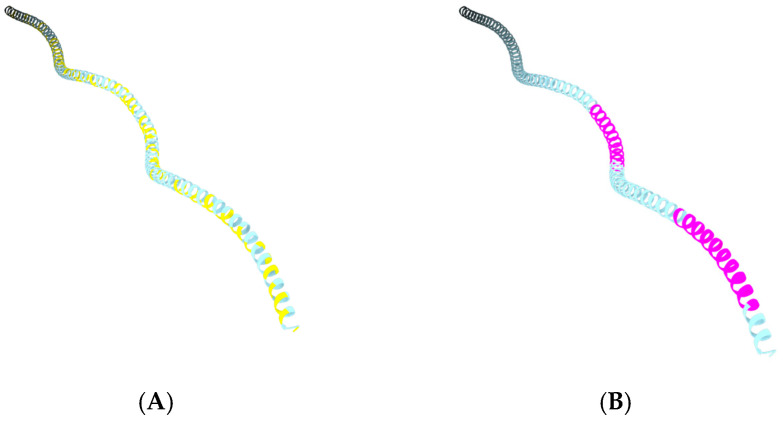
The “Coiled-Coil Rigidity” architecture of tropomyosin (Der p 10). (**A**) The Superhelical Scaffold: Three-dimensional model of the Der p 10 homodimer (steel blue) based on UniProt entry P06753. The Hydrophobic Zipper: Highlighted residues in yellow correspond to the *a* and *d* positions of the heptad repeat. This internal interface “zips” the helices together, providing the thermal stability required for the protein to survive as a food-matrix survivor. (**B**) Conserved Epitope Landscape: Mapping of the major linear IgE-binding epitopes (magenta). The image illustrates how the rigid supercoil preserves these motifs (e.g., LERTEERA) across processing conditions, maintaining cross-reactivity between mites and shellfish. The models were generated using AlphaFold (Entry P06753) and visualized in UCSF ChimeraX. Note: Reflecting its unbroken, highly uniform coiled-coil α-helical architecture, the computational model exhibits an overall average pLDDT score of 94.75. Key to amino acid abbreviations (in the caption of the figures of structures): A: Alanine; D: Aspartic Acid; E: Glutamic Acid; F: Phenylalanine; G: Glycine; H: Histidine; I: Isoleucine; K: Lysine; L: Leucine; N: Asparagine; P: Proline; P(OH): Hydroxyproline; Q: Glutamine; R: Arginine; S: Serine; T: Threonine; V: Valine; W: Tryptophan; Y: Tyrosine; X: any residue.

Tropomyosins from crustaceans, mollusks and some fish share a parallel α-helical coiled-coil architecture that supports conserved IgE epitopes and broad cross-reactivity across invertebrate species [137,138,139]. Structural and modeling studies consistently depict an elongated coiled-coil along most of the molecule, stabilized by characteristic (a–b–c–d–e–f–g)\ₙ heptad repeats with hydrophobic residues at positions a and d, which form an inter-helical interface [139]. This superhelical rigidity is reflected in their high α-helix contents and contributes to proteolytic and thermal stability, allowing tropomyosins to act as “supercoil” allergens [137,140] (Figure 4).

Epitope mapping across shrimp, fish and molluscan tropomyosins has identified multiple linear B-cell epitopes and conserved T-cell epitopes distributed along the coiled-coil [77,140,141,142]. In fish–shellfish comparisons, conserved short motifs, such as LERTEERA and LKTVQNN, and critical residues, including E, A, L and R, co-segregate with cross-reactive IgE binding and basophil activation despite only moderate global identity [142,143,144,145,146,147,148]. Detailed analyses in oyster and mantis shrimp show 7–10 linear IgE epitopes and several conformational mimotopes, together with multiple conserved CD4^+^ T-cell epitopes that support strong proliferation and cross-species reactivity [77,141,142]. Clinical and serologic data confirm that these conserved epitope regions underlie the extensive cross-reactivity among crustaceans, mollusks, mites and insects, and can generate asymptomatic shellfish sensitization in mite-sensitized patients [78,137,138].

Processing perturbs but does not necessarily abolish this algorithm. Reviews and experimental work indicate that thermal or high-pressure treatments, cold plasma, ultrasound, glycation and especially enzymatic hydrolysis can decrease the α-helix content, increase random coils and reduce IgE binding, sometimes by 40–70% [137,140]. However, structural modeling and degradation studies show that overall stability and coiled-coil integrity, more than raw sequence identity, govern endolysosomal peptide generation and T-cell-epitope display [137,139]. Highly conserved B- and T-cell epitope regions are often retained across species and treatments [77,139,141]. Tropomyosins therefore exemplify a “Coiled-Coil Rigidity” architecture: a superhelical scaffold that preserves linear and conformational epitopes across invertebrates, with allergenicity meaningfully reduced only when this coiled-coil is substantially unwound or fragmented [77,78,137,141,142].

### 5.6. Repetitive Motif Persistence: The Wheat Tri a 19 “Redundant Motif”

Tri a 19 (ω-5 gliadin) represents a cereal-grain implementation of structural redundancy. As an alcohol-soluble prolamin storage protein, ω-5 gliadin is rich in glutamine and proline and organized into highly repetitive domains [80,149]. Peptide-array mapping in WDEIA patients has defined seven key IgE-binding epitopes—QQIPQQQ, QQLPQQQ, QQFPQQQ, QQSPEQQ, QQSPQQQ, QQYPQQQ and PYPP—of which the QQXPQQQ/QQSPEQQ motifs are immunodominant [149,150,151]. Mutational analysis of QQIPQQQ and QQFPQQQ shows that Gln at positions 1 and 5–7 and Pro at position 4 are critical, yielding a consensus QXXPQQQ motif. Related motifs such as QQFHQQQ, QSPEQQQ, YQQYPQQ and QQPPQQ further expand the epitope repertoire [149,152].

**Figure 5:**
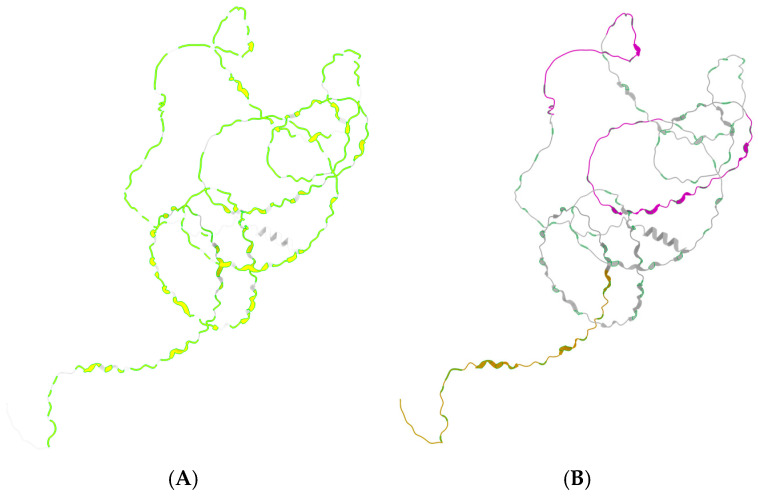
Repetitive redundancy and intrinsic disorder of Tri a 19 (ω-5 gliadin). (**A**) The Sequence-Level Cage: 3D model of the intrinsically disordered ω-5 gliadin scaffold. Yellow highlights denote the high density of Proline and Glutamine residues, which create a slippery, protease-resistant “chemical shield” despite the absence of a fixed 3D fold. (**B**) The Multivalent Epitope Grid: Mapping of immunodominant repetitive motifs (magenta), such as the QQXPQQQ consensus sequence. The repetitive nature of these sites ensures that even after partial proteolytic cleavage, multiple independent IgE-docking sites survive to trigger clinical reactions. The models were generated using AlphaFold (Entry Q402I5) and visualized in UCSF ChimeraX. Note: Because prolamins are characteristically non-globular, polymorphic and highly prone to concentration-dependent aggregation in solution, this static 3D model is presented strictly as an exploratory, high-entropy ensemble approximation rather than an experimentally validated native physiological fold (see Section 6 for full discussion). Reflecting its lack of a fixed, globular architecture, the computational model exhibits a very low overall average pLDDT score of 31.33. Key to amino acid abbreviations in Figure 4 caption.

These Q/P/Q-rich motifs occur at very high copy numbers in ω-5 gliadin and in related gliadins and glutenins. For example, a single ω-5 gliadin may contain >20 copies of QQFPQQQ and multiple copies of QQIPQQQ, many of which are overlapping [93]. This dense repetition creates a “sequence-level cage” in which the functional survivor unit is a short peptide repeat rather than a complex 3D fold. Similar QQXPQQQ-like motifs in γ-gliadins and low-molecular-weight glutenins cross-react with ω-5 gliadin-specific IgE and likely contribute to the severity and breadth of reactions [80,149] (Figure 5).

Functional digestion studies show that pepsin or pepsin–trypsin treatment of purified ω-5 gliadin generates peptides that retain strong IgE binding, and that subsequent tissue-transglutaminase (tTG) treatment cross-links glutamine residues within and between these peptides. The resulting high-molecular-mass aggregates (≈40 –> 200 kDa) exhibit significantly enhanced IgE binding and skin-prick reactivity compared with intact ω-5 gliadin [149,153,154]. While the precise contribution of proline content to protease resistance is inferred from the prolamin-like sequence and not quantified in these studies, the persistence and cross-linking of Q- and P-rich epitope peptides strongly support a protease-resistant, multivalent epitope grid [80,149].

Clinically, Tri a 19 is a major biomarker for WDEIA and adult-onset wheat allergy. Specific IgE to ω-5 gliadin shows higher diagnostic accuracy for WDEIA than wheat-sIgE alone and captures cases that would be missed by whole-extract testing [155,156,157]. Large clinical series indicate that a majority (often ~80%) of WDEIA patients have IgE to ω-5 gliadin, with the remainder frequently recognizing high-molecular-weight glutenin. Combined measurement of Tri a 19 with HMW glutenin and selected γ-gliadin epitopes substantially improves sensitivity and specificity, especially when incorporated into epitope-resolved assays [92,155,156,158,159,160,161].

Taken together, Tri a 19 exemplifies a “repetitive redundancy” architecture: densely packed, protease-resistant QXXPQQQ-type motifs generate multiple, independently functional IgE docking sites that survive digestion and can be reorganized—via host tTG and other factors—into highly potent supramolecular allergens under physiological stress [79,80,149,152,154].

Taken together, the structural archetypes examined throughout this study reveal a biophysical continuum: gastrointestinal survival can be achieved either through absolute conformational rigidity at one extreme or complete entropic flexibility at the other. Table 2 provides a final, formalized classification of these diverse structural paradigms, detailing how distinct molecular frameworks govern the persistence of the “Survivor Peptide” landscape.

### 5.7. Biophysical Boundaries: The Selective Biological Filter of the Gut–Mammary Axis

A critical conceptual paradox emerges when matching our proposed ‘stability architectures’ with broader epidemiological profiles: if structural resilience alone governs allergenicity, why do certain ultra-stable protein classes appear so selectively in maternal breast milk? Structural resilience alone does not govern which food allergens appear in human breast milk. Digestion-resistant dietary proteins such as cow’s milk caseins and β-lactoglobulin, egg ovalbumin and ovomucoid, wheat gliadins, and peanut Ara h 2/6 are reproducibly detected in human milk, typically at low ng/mL levels, with substantial intra- and inter-individual variability [8,11,12,17,29,118,162,163].

Modern liquid chromatography–tandem mass spectrometry (LC-MS/MS) profiling demonstrates that these dietary antigens appear predominantly as digestion-derived peptides and structured fragments, whereas the presence of intact native macromolecules is rarely or inconsistently confirmed, establishing a necessary paradigm shift from an “intact protein” to a “peptide-centric” perspective [8,10,11,163,164].

This biophysical divergence carries profound clinical implications as breastfeeding and formula feeding expose infants to similar dietary proteins but within very different immunological and nutritional contexts. Clinical cohorts and trials in cow’s milk protein allergy (CMPA) consistently show that feeding pattern and formula type influence both the development and resolution of allergy. In a retrospective CMPA cohort comparing six therapeutic diets, breastfed infants had the highest probability and fastest acquisition of tolerance to cow’s milk protein. Soy and extensively hydrolyzed formulas were associated with a markedly lower likelihood of tolerance and longer time to tolerance, despite adequate growth on hydrolysates [165]. In non-IgE-mediated CMPA with gastrointestinal symptoms, prior exposure to standard cow’s milk formula before symptom onset predicted later tolerance on follow-up oral food challenge [166]. Similarly, in food protein-induced allergic proctocolitis (FPIAP), any cow’s milk-based formula use during infancy was an independent predictor of delayed tolerance [167] and amino acid-based formulas were associated with later resolution compared with less-severe phenotypes [168]. Among formula-fed CMPA infants, a large multicenter cohort found that extensively hydrolyzed casein formula, especially when combined with *Lactobacillus rhamnosus* GG, induced higher 12-month tolerance rates (up to 78.9%) than rice, soy or amino-acid formulas, with long-term follow-up showing fewer later atopic manifestations and faster tolerance with this regimen [169]. At the population level, lower breastfeeding rates and higher formula use were associated with higher CMPA prevalence in Chinese infants [170] and a randomized trial showed that avoiding early cow’s milk formula supplementation reduced later cow’s milk sensitization and food allergy [171].

This discrepancy indicates that resistance to gastrointestinal degradation is a necessary but insufficient prerequisite for maternal–neonatal transmission: the gut–mammary axis operates as a multi-step, intestine–breast selective biological filter [14,29]. Within this axis, overall antigen transport depends heavily on a combination of host-specific parameters, including maternal digestion efficacy, intestinal and mammary epithelial barrier permeability, lactation stage, food-matrix protection, and maternal atopic status—the latter of which has been associated with elevated allergen or peptide shedding in several cohorts [8,11,12,17,30,164].

Maternal IgG is actively transferred to the fetus and nursing neonate and shapes early-life protection, tolerance and risk of immunopathology. Central to these processes is the neonatal Fc receptor (FcRn), which binds IgG in a pH-dependent manner and mediates both placental transport and regulation of IgG half-life [1,2,172,173,174].

During human pregnancy, IgG is taken up by syncytiotrophoblasts and transcytosed across the placenta via FcRn. Loss of FcRn binding (H435A mutation) markedly impairs ex vivo placental transfer, establishing FcRn as essential for maternofetal IgG delivery [2,172,174]. Mouse and humanized models further show that FcRn, but not Fcγ receptors, is the dominant driver of transplacental IgG transport and IgG engineered for enhanced FcRn affinity accumulates more efficiently in the fetus [173,175]. FcRn is also expressed in the mammary gland and neonatal intestine, mediating bidirectional IgG transport and recycling [1,2,173,176].

These transport pathways have functional consequences for allergies. Maternal allergen-specific IgG and IgG immune complexes (IgG-IC) transferred via FcRn in breast milk induce regulatory T cells and robust food allergen-specific tolerance in offspring [11,177]. In murine asthma models, FcRn-dependent acquisition of maternal allergen-specific IgG protects against allergic airway inflammation, whereas FcRn deficiency abrogates this protection [178,179,180]. Conversely, FcRn can also shuttle pathogenic complexes: IgE does not bind FcRn directly, but maternal IgE can reach the fetus within IgG anti-IgE/IgE immune complexes that bind and traverse FcRn-expressing epithelia, explaining why cord blood IgE is largely in complexed form and indicating that it potentially contributes to prenatal allergic sensitization [181,182,183].

Moreover, robust rodent and humanized neonatal models demonstrate that the presence of free milk-borne antigens alone is entirely insufficient for immunological protection; instead, the capacity of these fragments to safely prime neonatal tolerance critically depends on their packaging into maternal IgG–allergen immune complexes that exploit active neonatal FcRn receptor-mediated transcytosis and specialized antigen presentation to safely induce regulatory T (Treg) cells [14,29,32,39,177]. Consequently, the limited and specific set of dietary antigens characterized so far in breast milk surveys defines the precise physiological boundaries where intrinsic structural stability architectures intersect with host-specific transport mechanics, immune complex packaging, and mucosal barrier dynamics, rather than invalidating the baseline Survivor Peptide framework.

## 6. Limitations

While this review provides a comprehensive framework for allergen persistence, several limitations must be acknowledged. The in silico structural analysis relies on static representations which may not fully capture the dynamic conformational fluctuations occurring during the varying pH levels and enzymatic pressures encountered by allergens during maternal digestion. Furthermore, a major, explicit limitation of this framework centers on the complete isolation of our computational evaluations from real-world industrial processing and complex food matrix interactions. In native human diets, allergens are rarely ingested as pure, isolated proteins. Instead, their structural resilience, gastrointestinal degradation velocities, and subsequent entero-mammary translocation rates are profoundly modulated by matrix-level phenomena. These include the thermal generation of covalent Maillard-type glycation products, structural compaction or steric shielding within lipid-dense microenvironments (such as egg yolk or hydrophobic peanut lipid matrices) and mineral-dense micellar sequestration (such as milk caseins). These highly complex matrix configurations can either physically shield immunodominant epitopes from digestive proteases or, conversely, accelerate structural unfolding due to localized thermal destabilization. Because our current computational models evaluate isolated, static protein topologies, they cannot fully account for these processing-induced structural modifications or matrix-driven protection kinetics, representing a critical boundary condition for our conclusions. Finally, the translocation of allergens into breast milk is characterized by high inter-individual variability driven by maternal gut permeability, allergy status, and individual digestion kinetics, suggesting that structural resilience is a necessary but not the sole determinant of antigen presence in milk.

A notable methodological limitation of the structural framework proposed in this review centers on the varying predictive accuracy of modern computational tools across distinct allergen archetypes. While advanced deep-learning frameworks (e.g., AlphaFold2) excel at mapping rigid, well-folded globular proteins, they present severe pitfalls when applied to intrinsically disordered regions or highly heterogeneous protein targets, highlighting a systemic need to transition from static snapshots to dynamic conformational ensembles [184,185].

This computational constraint is highly relevant to cereal prolamins. Rather than adopting a single, fixed native state, prolamins display immense structural polymorphism in solution, dynamically shifting across globular, rod-like and highly extended ‘worm-like’ geometries [186]. Furthermore, molecular dynamics simulations demonstrate that prolamins undergo rapid structural unfolding, loss of native secondary structure elements and spontaneous fibrillar or particle transitions when exposed to processing conditions, electrical fields or microenvironmental interfaces [186,187]. Because mainstream deep-learning predictors fundamentally rely on a static-fold assumption, they cannot fully encapsulate this environment-dependent structural flexibility. Consequently, while computational pipelines offer indispensable exploratory value, the static 3D configurations of prolamins presented in this manuscript (such as Tri a 19) must be interpreted strictly as high-entropy ensemble approximations rather than definitive, native physiological folds.

## 7. Conclusions

In summary, this review suggests that certain food proteins are more likely to appear in breast milk and promote tolerance because their structurally resilient architectures allow them to survive maternal digestion in an immunologically intact form. Identifying these structural stability characteristics offers a useful conceptual model for understanding how specific dietary antigens persist through maternal digestion and navigate the maternal–neonatal interface. This structural framework has potential translation for refining component-resolved diagnostics (CRD) to improve diagnostic precision, assist in risk stratification, and help distinguish genuine clinical sensitization from cross-reactivity [188,189,190,191]. Rather than relying on crude food extracts that contain variable mixtures of labile components, interpreting diagnostic panels through the lens of digestion-resistant macromolecular archetypes, such as 2S albumins (Ara h 2/6) or casein-type structures (Bos d 4–8) that conserve immunoreactive cores and IgE-binding epitopes despite high temperatures and physiological breakdown, can assist clinical practitioners in predicting severe reactions and anaphylaxis risk [116,192,193,194,195,196]. Crucially, the practical relevance of this structural model remains directly bounded to the selective set of food families currently documented within human breast milk survey profiling, specifically major cow’s milk, hen’s egg, peanut and fish allergens [8,11,12,30,31,51,118]. Furthermore, this framework acknowledges that clinical allergenicity is not driven by static macromolecular stability alone; rather, it exists as a dynamic spectrum where surviving linear sequences serve as early structural templates for primary gut sensitization, while extrinsic processing modifications and complex food matrices heavily govern downstream epitope availability.

From a nutritional and preventative standpoint, this model helps contextualize why routine maternal restriction or blanket food exclusion diets during pregnancy and lactation are widely discouraged in contemporary clinical guidelines [31,34,195,197,198]. Except in instances of verified maternal or infant clinical allergy, a continued, diverse maternal diet during lactation appears consistent with normal physiologic pathways of tolerance induction. This varied maternal intake permits the steady, low-dose (ng/mL) passage of structurally resilient dietary fragments or digestion-derived peptides into human milk, where their packaging into maternal IgG/IgA immune complexes, alongside native tolerogenic milk factors, may contribute to mucosal and systemic immune education in the breastfed offspring [8,30,31,34,38,118,195].

Ultimately, the “Survivor Peptide” hypothesis is intended as an integrative conceptual framework, offering distinct avenues of utility across three translational tiers. For basic and clinical researchers, it provides a baseline blueprint to guide the design of future in vitro digestion assays and animal trials, specifically testing how distinct epitope architectures, peptide fragments and maternal immune complex densities modulate neonatal FoxP3^+^ regulatory T (Treg) cell responses, an area where detailed molecular mechanisms and structural determinants are still actively emerging [11,38,51,116,199]. Importantly, future experimental configurations must transition from isolated protein models to dynamic testing environments that account for industrial processing chemistry and food-matrix-mediated protection. For clinical practitioners, it offers a structural rationale to support cautious, evidence-based interpretation of CRD panels and severity risk stratification, balancing the distinct roles of stable conformational folds against persistent linear fragments while fully respecting the inherent limitations of molecular diagnostics, including cost, panel constraints and the reality that CRD can augment but never replace the oral food challenge (OFC), which remains the diagnostic gold standard [188,190,193,195,197,200]. For breastfeeding mothers, it provides a reassuring, non-prescriptive mechanistic explanation for current infant feeding guidelines, illustrating how maintaining a standard, varied maternal diet supports the natural transmission of tolerogenic immune signals to their infant without the need for unnecessary, stressful dietary restrictions [31,34,38,195,197].

## Figures and Tables

**Table 1:** Molecular Resilience Architectures: a comparative framework of structural stability, digestive persistence and clinical outcomes for major food allergens.

Allergen	Molecular Features and Structure	Allergenic/ Epitope Information	Processing and Digestive Stability	Notes for Scoping Review	Sources
Gal d 1— Ovomucoid	186 aa, 3 tandem domains (Gal d 1.1–1.3); highly glycosylated; rigid tertiary structure by NMR; thermally stable in MD simulations	Multiple linear IgE/IgG epitopes (9 IgE, 8 IgG) mapped by overlapping peptides; conformational epitopes important, IgE binding reduced after reduction/denaturation	Thermally stable; structural changes under combined heat + electric field; reduction disrupts conformational epitopes and decreases IgE binding	Major egg-white allergen; strong association of sequential epitopes with persistent allergy and baked-egg reactivity	[70,71,72,73,74,75]
Gal d 2— Ovalbumin	Globular phosphoglycoprotein; heterogeneous mixture of >130 proteoforms due to PTMs (phosphorylation, acetylation, oxidation, glycosylation)	Five dominant IgE epitopes mapped: L38–T49, D95–A102, E191–V200, V243–E248, G251–N260	PTMs alter IgE/IgG binding: more heavily modified forms show reduced binding; sialylated glycoforms show highest binding	Major allergen; structural heterogeneity and defined sequential epitopes make it a key model for structure–allergenicity relationships	[71,76,77]
Ara h 2 (2S albumin)	Disulfide-stabilized 2S albumin with four disulfide bridges; repeated DPYSP(OH)S motifs; proline hydroxylation is critical PTM	Both conformational and linear epitopes; single hydroxylated DPYSP(OH)S-containing peptide recapitulates major linear epitope; small hydroxylated peptides (15–27 aa) trigger degranulation	In gastric digests of whole peanut, Ara h 2 and Ara h 6 are largely intact; SDRPs from Ara h 2 strongly inhibit IgE and are epitope-rich; matrix digestion yields mixtures where 2S albumins dominate allergenicity	IgE to Ara h 2 strongly predicts clinical reactivity; diagnostic accuracy depends on preserving hydroxyprolines; Ara h 2- and Ara h 6-specific IgE are mostly non-cross-reactive, arguing for separate measurement; structural stability supports potent, persistent allergy	[78,79,80,81,82]
Bos d: β-LG, α-LA, caseins	β-LG: lipocalin β-barrel with ligand-binding calyx; α-LA, serum albumin: globular; caseins: phosphorylated IDPs in micelles; heat/Maillard glycation alters folding and aggregation	Many linear and conformational epitopes on β-LG, α-LA, α-caseins; stable β-LG epitopes: 92–100, 125–135/138, 149–162; stable α-LA epitopes: 63–79, 80–93; stable α-casein epitopes: 25–32, 84–90, 125–132; conformational hotspots in αs1-casein	Industrial processing induces aggregation/denaturation but digestion of milk/dairy releases 3–5 kDa SDRPs (≈10 aa), mostly from caseins, which have overlapping IgE epitopes; SDRPs aggregate, cross-inhibit IgE and provoke skin responses; heat-treated whey initially less IgE-reactive but later digests show more linear epitopes	Major infant allergen; epitope-rich SDRPs and processing-enhanced linear epitopes support persistent CMA; epitope-level and component diagnostics must consider matrix and processing; cross-reactivity with other mammalian milks	[83,84,85,86,87]
Tropomyosins (shellfish/ invertebrates)	Parallel α-helical coiled-coil dimers; thermostability varies (Tm: ~33–63 °C) and shapes degradation; some processing introduces glycation/structural loosening	Multiple linear B-cell/IgE epitopes mapped (e.g., shrimp: 47–61, 97–108, 244–257) with degranulation; several T-cell epitopes; many epitope regions conserved across shrimp, clams and other shellfish; T-cell cross-reactivity more limited	Highly thermostable; can withstand common cooking methods; structural stability drives slower endolysosomal degradation and distinct peptide repertoires; high-pressure/ultrasound/glycation reduce α-helix content and IgE binding but leave some recognizable linear epitopes after digestion	Major crustacean allergen; epitope-defined TM improves molecular diagnosis and may guide peptide immunotherapy; shared epitopes explain broad IgE cross-reactivity and asymptomatic shellfish sensitization in mite-sensitized patients; processing can partly reduce but rarely abolish allergenicity	[88,89,90,91]
Tri a 19 (ω-5 gliadin)	Alcohol-soluble, highly repetitive proline/glutamine-rich gluten protein; encoded on 1B and 1D with multiple omega-5 gliadins harboring WDEIA epitopes	Multiple repeated linear IgE epitopes across repetitive domains; genetic deletion of 1B omega-5 gliadins reduces, but does not eliminate, IgE reactivity because additional omega-5 gliadins on 1D carry similar epitopes	Repetitive structure confers protease resistance to epitope-containing peptides; breeding or biotechnology removing omega-5 gliadins lowers IgE reactivity; detailed digestion fragments not specified in these excerpts	Key molecular biomarker for IgE-mediated wheat allergy and WDEIA; rTri a 19 is widely used in component-resolved diagnostics; BAT with ω-5 gliadin refines diagnosis and reduces OFC need; less central for baker’s asthma, where α-amylase inhibitors dominate	[92,93,94]

Abbreviations: aa, amino acids; α-LA, α-lactalbumin; BAT, basophil activation test; β-LG, β-lactoglobulin; CMA, cow’s milk allergy; Dx, diagnosis; IDP, intrinsically disordered protein; IgE, immunoglobulin E; IgG, immunoglobulin G; MD, molecular dynamics; NMR, nuclear magnetic resonance; OFC, oral food challenge; PTM, post-translational modification; SDRP, short digestion-resistant peptide; Tm, melting temperature; and WDEIA, wheat-dependent exercise-induced anaphylaxis.

**Table 2:** Structural characteristics, thermal stability and aggregation properties of proteins within the proposed Stability Gradient.

Architecture Category	Formalized Biophysical Definition	Representative Allergen Member	Key Secondary Structure Environment	Disulfide Bond Metrics (Per Domain/Unit)	Food Processing and Thermal Susceptibility	Quaternary Aggregation Propensity
“Covalent Cage”	Intramolecular shielding of linear or conformational epitopes, reinforced by a dense network of covalent cross-links that prevent global chain separation during proteolysis.	Gal d 1 (Ovomucoid) Ara h 2 (2S Albumin)	Variable; accommodates dense, rigid loops (Gal d 1) or compact α-helical bundles (Ara h 2)	High Density: 3 disulfide bonds per domain (Gal d 1) 4 disulfide bonds (Ara h 2)	Extremely Resilient: Maintains IgE-binding capacity after prolonged boiling; resists complete gastric proteolysis	High: Propensity to form highly stable homodimers or complex macro-aggregates that shield inner loops.
The Topological Shield	Extended, dense carbohydrate clustering (*N*- or *O*-glycosylation) that creates a localized steric and electrostatic hydration barrier, physically masking the underlying peptide backbone from protease docking.	Gal d 6 (YGP42)	Mixed α- and β-core scaffold; relies on highly exposed surface loops where glycan trees anchor	Variable/Secondary: Disulfide bonds are secondary to the density of covalent carbohydrate attachments	Modulated by Processing: Susceptible to high dry heat; Maillard reactions can either destroy glycans or form advanced glycation end-products.	Medium: Glycan-mediated stabilization primarily prevents unfolding rather than driving non-specific self-aggregation.
“Redundant Scaffold” and Reversible Memory	Enclosure of internal hydrophobic cores or ligand-binding calyces within a rigid secondary structural assembly to minimize protease docking.	Bos d 5 (β-Lactoglobulin)	β-Sheet Dominant: Anti-parallel β-barrel central matrix	Low to Moderate: 2 intact intramolecular disulfide bonds (Bos d 5)	Thermally Sensitive: Denatures under high wet heat, unzipping the calyx and exposing previously hidden cryptic epitopes	High: Exhibits distinct pH-dependent dimerization and concentration-dependent oligomerization.
“Coiled-Coil Rigidity”	Supercoiled, parallel or anti-parallel alpha-helices characterized by a conserved heptad repeat pattern that provides longitudinal mechanical resistance that prevents enzymatic unzipping.	Der p 10 (Tropomyosin)	α-Helix Dominant: Nearly 100% extended α-helical coiled-coil rod morphology	Absent: Relies entirely on hydrophobic “knobs-into-holes” packing along the helical interface rather than covalent bonds	Resilient to Boiling: Heat induces transient denaturation, but the highly conserved heptad pattern allows for spontaneous, perfect renaturation upon cooling.	Low to Medium: Forms stable lateral filamentous polymer chains but low amorphous macro-aggregation.
“Persistent Repetitive Motifs”	Extended, intrinsically unstructured poly-amino acid stretches (e.g., poly-glutamine) lacking standard endoprotease consensus cleavage sites that drive macro-molecular network formation.	Tri a 19 ω-5 Gliadin	Disordered/Random Coil: Lacks defined classical secondary structures; dominated by repetitive beta-turns and coils	Absent in repetitive domain: Lacks covalent caps, relying instead on dense intermolecular networks	Highly Processed Modulated: Baking and cross-linking (e.g., transglutaminase) stabilize gluten networks, increasing mucosal persistence.	Very High: Drives extensive hydrophobic coacervation and liquid–liquid phase separation into protective protein matrices.

## Data Availability

No new data were created or analyzed in this study.

## Abbreviations

3D	Three-dimensional
α-LA	α-lactalbumin (Bos d 4)
β-LG	β-lactoglobulin (Bos d 5)
aa	Amino acids
APC	Antigen-presenting cell
Ara h 2	Arachis hypogaea allergen 2 (peanut 2S albumin)
Arg	Arginine
Asp	Aspartic Acid
BAT	Basophil activation test
Bos d	Bos domesticus (bovine milk allergens)
B-cell	Bone marrow-derived lymphocyte
CCL28	C-C motif chemokine ligand 28
CCR10	C-C motif chemokine receptor 10
CD23	Low-affinity immunoglobulin E receptor (cluster of differentiation 23)
CD4^+^	Cluster of differentiation 4-positive (helper T cells)
CDR	Complementarity-determining region
CMA	Cow’s milk allergy
CRD	Component-resolved diagnostics
D104	Aspartic acid at position 104
DPYSP(OH)S	Peptide motif containing hydroxyproline
Dx	Diagnosis
ELISA	Enzyme-linked immunosorbent assay
FcεRI	High-affinity immunoglobulin E receptor
FcRn	Neonatal Fc receptor
Fcγ	Fc-gamma receptor (immunoglobulin G receptor)
FoxP3^+^	Forkhead box P3-positive (lineage marker for regulatory T cells)
FPIAP	Food protein-induced allergic proctocolitis
FPIES	Food protein-induced enterocolitis syndrome
Gal d 1	Gallus domesticus allergen 1 (ovomucoid)
Gal d 2	Gallus domesticus allergen 2 (ovalbumin)
Gal d 6	Gallus domesticus allergen 6 (yolk vitellogenin fragment)
GI	Gastrointestinal
Glu	Glutamic acid
HMW	High molecular weight
IDP	Intrinsically disordered protein
IgA	Immunoglobulin A
IgE	Immunoglobulin E
IgG	Immunoglobulin G
IgGIC	Maternal immunoglobulin G-allergen immune complex
IL-4	Interleukin-4
IL-13	Interleukin-13
KD	Dissociation constant
kDa	Kilodalton
LC–MS/MS	Liquid chromatography with tandem mass spectrometry
Lys	Lysine
MD	Molecular dynamics
MHC II	Major Histocompatibility Complex class II
NMR	Nuclear magnetic resonance
OFC	Oral food challenge
PDB	Protein Data Bank
pg/mL	Picograms per milliliter
ng/mL	Nanograms per milliliter
pLDDT	Predicted Local Distance Difference Test (AlphaFold structural confidence metric)
PTM	Post-translation modification
Q139	Glutamine at position 139
RCSB	Research Collaboratory for Structural Bioinformatics
RMSD	Root mean square deviation
RMSF	Root mean square fluctuation
SASA	Solvent-accessible surface area
SDRP	Short digestion-resistant peptide
sIgE	Specific immunoglobulin E
S–S	Disulfide bond
T-cell	Thymus-derived lymphocyte
TGF-β	Transforming growth factor-beta
Th2	T helper type 2
Tm	Melting temperature
Treg	Regulatory T cell
Tri a 19	Triticum aestivum allergen 19 (ω-5 gliadin)
tTG	Tissue transglutaminase
UCSF	University of California, San Francisco
UHT	Ultra-high temperature
UniProt	Universal Protein Resource database
WDEIA	Wheat-dependent exercise-induced anaphylaxis
YGP40	Yolk glycoprotein 40
YGP42	Yolk glycoprotein 42
Å	Angstrom (10^−10^ m, structural distance metric)
